# Physical assessment competencies for nurses: A quality improvement initiative

**DOI:** 10.1111/nuf.12725

**Published:** 2022-04-17

**Authors:** Nicole M. Fontenot, Shannan K. Hamlin, Steven J. Hooker, Theresa Vazquez, Hsin‐Mei Chen

**Affiliations:** ^1^ Department of Nursing, Center for Nursing Research, Education and Practice Houston Methodist Academic Institute Houston Texas USA

**Keywords:** clinical deterioration, failure to rescue, physical assessment, quality improvement

## Abstract

As the only healthcare providers caring for hospitalized patients every hour of every day, nurses have a responsibility to keep patients safe. Physical assessment is a basic but essential nursing skill that fosters patient safety. Assessing a patient's current status enables nurses to recognize early patient deterioration. Contemporary nursing practice relies on vital signs and technology to aid in the detection of patient deterioration. The aim is to describe the Methodist Proficient Assessment Competency (MPAC^©^) quality improvement initiative. Surveys and directly observed patient assessment data were used to evaluate attitudes and practices. One hundred and seventy‐nine pre‐MPAC audits were conducted, followed by 1391 post‐MPAC audits. Pre‐ compared with post‐MPAC audits showed significant improvements in complete physical assessments (78% vs. 94%; *p* < .001), timeliness (within 4 h; 64% vs. 91%; *p* < .001) and accuracy (67% vs. 95%; *p* < .001) of documentation. In conclusion, nurses have a responsibility to quickly identify changes in a patient's condition and intervene to prevent serious adverse events. Taking the needed time to perform a full physical assessment at the beginning of the shift along with timely and accurate documentation, allows nurses to acquire the knowledge they need to establish a patient's current clinical status and usual behaviors, thereby facilitating early recognition of subtle changes that could indicate deterioration.

## 1 INTRODUCTION

Physical assessment is a crucial nursing skill. However, as nurses are tasked with other skills this aspect of nursing may be less practiced. As turnover in an inpatient setting increased with more newly licensed registered nurses as well as nursing management turnover, thought leaders at a large academic medical center identified a worrying trend in the nursing workforce around the performance of a full (head‐to‐toe) physical assessment. From 2014 to 2017, the number of Rapid Response Team (RRT) activation calls increased with greater time delays in calling the RRT, which led to worse patient deterioration and outcomes. Members of the RRT, all of whom are nurse practitioners, noticed that nurses knew less about their patients with a wide range of variation in nursing practices from unit to unit.

In 2015, the nurse education department leadership and the nurse practitioner leadership began to investigate and develop mitigation strategies for improving nurse's clinical knowledge of their patients to detect early patient deterioration. During an education fair that year, the nurse practitioners performed simulated rapid response scenarios with nurses from departments with the highest number of RRT calls; many nurses skipped any type of physical assessment with few nursing interventions before the RRT arrived. From this exercise, the leaders in the nursing education department decided that refocusing nurses on the basic practice of physical assessment may improve early detection of patient deterioration and improve patient outcomes.

Methodist Proficient Assessment Competency (MPAC^©^) was created in 2017. The goal for MPAC was to conduct a quality improvement project to assess the current practice of physical assessments, create a standardized, full physical assessment incorporating all body systems, provide education to all inpatient nurses, and complete follow‐up audits to determine whether nursing practice not only changed immediately after the education but was sustained. The aim of this study is to describe the MPAC quality improvement initiative along with outcomes and implications for nursing practice.

## 2 BACKGROUND

Physical assessment is a basic but essential nursing skill. Being able to assess the patient's current condition can help identify early changes. Knowledge of a patient's clinical status and usual behaviors gained through a full (head‐to‐toe) physical assessment is a key influence on a nurse's ability to recognize subtle changes in a patient's condition.^1^, ^2^, ^3^, ^4^ The importance of early recognition of deterioration before overt physiologic signs, such as vital sign changes, cannot be overstated given the link between unrecognized patient deterioration and serious adverse events.^5^, ^6^, ^7^, ^8^, ^9^ However, physical assessment as practiced daily in contemporary nursing focuses more on vital signs than physical assessment; this is likely due to time restraints and a reliance on technology to determine patient's clinical status.^10^ Often nurses face barriers to completing a physical assessment, including lack of time and unit culture,^11^ ambiguity around who is responsible for physical assessments, reliance on technology, and lack of confidence in assessment skills.^12^, ^13^

Electronic warning systems (EWSs) and RRTs, which often depend on patient vital signs, have widespread acceptance as the only safety interventions for detecting patient deterioration. However, the detection of abnormal vital signs is an end‐stage deterioration, which may be detected earlier using a thorough patient assessment.^10^ Schnock et al.^13^ found that nursing documentation of their physical assessment often can predict patterns of patient deterioration events in both the critical care and acute care environments.^14^ Indeed, the EWS used at the hospital in which the aforementioned project was conducted, relies upon timely, accurate nursing physical assessment data and documentation to optimally function in detecting patient deterioration.

Before MPAC, no formal instruction on physical assessment was provided to nurses at the project site. Preceptors were expected to validate a newly hired nurse's skill and knowledge during orientation, but it was dependent on each individual preceptor to determine what was considered a full physical assessment. Additionally, most preceptors and even nurse managers expect nurses to be proficient in performing a physical assessment, so often unit‐based orientation is focused more on other tasks and skills. MPAC was implemented to help nurses recognize clinical deterioration through a standardized and systematic approach to physical assessment.

## 3 METHODS

MPAC was a quality improvement initiative undertaken at a large academic medical center in the southwest region of the United States. The hospital's Institutional Review Board determined no approval or oversight was required. The specific aims of the initiative were to improve completeness (head‐to‐toe) of physical assessments performed by inpatient nurses, improve timeliness (within 4 h of conducting an assessment) and accuracy (assessment components observed matched what was documented in the electronic health record [EHR]) of assessment documentation, and improve availability of necessary assessment equipment (i.e., penlight and stethoscope).

## 4 INTERVENTIONS

### 4.1 Pre‐MPAC

After completing a literature search, reviewing the Texas Nurse Practice Act, and the hospital's policy on assessment, a standardized physical assessment was established. Evidence shows the timeliness, accuracy, and relevancy of documented assessment findings increase with the use a structured patient assessment framework.^15^ Table 1 shows components of the MPAC‐standardized physical assessment. A booklet with the new standardized physical assessment was produced as a learning aid for MPAC participants. In addition, a 10 min video was created, which demonstrated the new standardized physical assessment.

**Table 1:** Components of the MPAC‐standardized physical assessment

Preparations for completing a physical assessment	Performs hand hygiene and dons personal protective equipment as needed
	Introduces self and confirms patient identification
	Has appropriate equipment for completing a physical assessment (penlight and stethoscope)
Performs a physical assessment	Assesses orientation and mental status
	Assesses delirium using an appropriate tool (CAM, CAM‐ICU, or 4AT)
	Assesses pupils
	Assesses skin, including back, sacrum, and any other bony prominences
	Assess extremities for sensation, strength, movement, pulses, and capillary refill
	Assess pain
	Assess edema
	Assess lung sounds on both the front and back
	Assesses heart sounds
	Assesses the abdomen (listens first, then palpate abdomen)
Documents a physical assessment	Documents physical assessment within 4 h
	Documented assessment matches assessment that was performed

Abbreviations: 4AT, 4 A's Test; CAM, Confusion Assessment Method; CAM‐ICU, Confusion Assessment Method‐intensive care unit; MPAC, Methodist Proficient Assessment Competency.

During this same time, we conducted a gap analysis to identify the current state of practice in physical assessments. First, an anonymous informal, nonvalidated survey was sent to all inpatient nurses asking them to list the barriers to completing a physical assessment. The survey showed that many of the nurses who responded (9% response rate) perceived other priorities such as administering medications, drawing labs, patient hygiene, and mobility were more urgent than completing a patient physical assessment or documenting it fully and accurately in the EHR.

A second anonymous informal, nonvalidated survey was sent to the nursing unit leadership, asking their attitudes regarding nurses conducting a full physical assessment and how important is it for nurses to recognize a change in a patient's condition. With a 32% response rate, 25% of nursing leaders reported it was “extremely unimportant” for nurses to conduct a full physical assessment and/or recognize a change in a patient's condition with some leaders commenting it was predominantly the physician's responsibility.

The second phase of the gap analysis was to evaluate whether nurses were currently conducting full physical assessments. During February and March 2018, pre‐MPAC audits were conducted by the nursing education department on all inpatient nursing units. The nurse educators arrived at nursing units at change of shift and randomly selected one to two nurses, to observe the nurse's initial physical assessment. Nurse evaluators were in the patient room with the nurse, while they were conducting a physical assessment. The nurse evaluators did not provide feedback to the nurse during their assessment, but would review with the nurses after they had left the patient room. Evaluators collected data on a standardized tool that mirrored the evidence‐based, standardized full physical assessment. These audits showed a large gap between best practice, hospital policy, and actual nursing practice.

### 4.2 MPAC initiative

After the pre‐MPAC audits were completed, the MPAC curriculum was developed by a team of nursing professional development specialists with a wide range of experience in critical care and medical‐surgical nursing. This curriculum consisted of didactic training about the importance of physical assessment, the new MPAC‐standardized physical assessment with a demonstration video, recognizing patient deterioration, and key nursing interventions to ameliorate deterioration. After the didactic lecture, three skills stations were designed so that nurses could demonstrate what they learned during the lecture. The first station was a return demonstration of the standardized physical assessment on a standardized patient. The second station was high‐fidelity simulation of a patient experiencing a clinical deterioration. The third station provided updated information about the hospital's EWS. Including simulation was essential, as it has been shown to improve patient safety.^16^

All inpatient nursing units were divided up into three groups and scheduled to attend in the spring, summer, and fall of 2018. Medical units completed the course first, as they had the most RRT calls each year. The medical units at this facility typically have a four or five to one patient to nurse staffing ratio. The medical units are broken up by patient population, with different units for pulmonary diseases, cardiology and heart failure, oncology and the bone marrow transplant units, infectious diseases and sepsis, renal diseases, general medicine, neurology, hepatology, and pretransplant liver patients. However, any medical unit can admit any type of medical patient if needed.

Surgical units attended in the fall of 2018. The surgical units that participated included the orthopedic units, general surgery, posttransplant, postcardiothoracic surgery, neurosurgery, head and neck surgery, and urological and gynecologic surgical units. These units generally have a four or five to one patient to nurse staffing ratio. During the week, when the operating rooms are busy, the surgical units are full of surgical patients but on the weekends, they typically fill up with medical overflow patients.

By the fall, nursing leadership requested that certain outpatient areas, such as the Emergency Department and the Postanesthesia Care Units, participate in the program as well. In October through December, these departments plus critical care, intermediate care, and Women's Services, attended MPAC. The critical care units include medical, surgical, transplant, cardiac, neurosurgical, and cardiovascular intensive care units. The intermediate care units take a mixture of medical and surgical patients who need more monitoring or mechanical ventilatory support than can be provided on the medical or surgical units. Staffing ratios in critical care are usually 2:1 patient to nurse ratio and 3:1 ratio in the intermediate care units.

Post‐MPAC audits were completed starting 1 month after each cohort finished their MPAC training. Using the same criteria and the same technique as the pre‐MPAC audits, nursing educators completed four to six audits per nursing unit per week for 4 weeks. Additionally, ongoing surveillance audits continued for each cohort for up to 12 months, to ensure the practice change was sustained. During the ongoing surveillance audits, the nurse educators modified the process to provide coaching when needed.

After the initial training was completed, MPAC was added to the nursing on‐boarding curriculum so all newly hired nurses receive training before their first day on their unit. Additionally, physical assessment competency checkoffs have been added to annual nursing competencies.

### 4.3 Measures and analysis

The key measures for this quality improvement project included:

- Completeness of the physical assessment
- Use of physical assessment equipment; specifically, penlight/pupilometers, and stethoscope
- Timeliness of documentation
- Accuracy of documentation

These four measures were chosen because of the primary gaps identified. Data were collected by the nurse educators as they performed their physical assessment audits and then compiled into a database. The data collection forms mirrored the standardized physical assessment (Table 1) and included the location, shift, and staffing ratio. Data were entered into an Excel™ database and STATA v16.1 for Windows^17^ analyzed the data. Percentage of completion, length of time to perform the physical assessment, assessment equipment utilized, timeliness of documentation, and accuracy of documentation were analyzed with two‐sample *T* tests with unequal variances.

## 5 RESULTS

Throughout the MPAC initiative, 1839 nurses (99.7% of the entire nursing staff) successfully completed the course. The pass rate for the first attempt at performing a physical assessment on a standardized patient was 88%. If a nurse did not pass on their first attempt, they could immediately try again after coaching from an educator; the pass rate for the second attempt was 87%. If they were not able to pass a second time, the nurse was required to repeat the course. Of those that repeated the course, 80% passed on their third attempt. Overall, five nurses were unable to successfully demonstrate the standardized physical assessment.

In total, 179 pre‐MPAC audits were performed on all 34 inpatient nursing units over a 3‐month period. Post‐MPAC audits (1391) were collected over an average of 10 months, starting 4 weeks after MPAC training concluded. Table 2 contains a complete summary of results.

**Table 2:** Comparison of MPAC outcomes by pre‐MPAC and post‐MPAC groups

	Pre‐MPAC *n* = 179	Post‐MPAC *n* = 1,391	*p*
Completeness of assessment			
Percentage of components completed	78%	94%	<.001
Equipment to perform assessment			
Used a penlight/pupilometer	87 (48.6%)	1202 (86.9%)	<.001
Used a stethoscope	166 (92.7%)	1379 (99.2%)	<.001
Timeliness of documentation			
Documentation completed within 4 h of assessment	116 (67.4%)	1205 (94.7%)	<.001
Accuracy of documentation			
Actual assessment corresponded to documentation	102 (64.2%)	1200 (90.6%)	<.001

Abbreviation: MPAC, Methodist Proficient Assessment Competency.

Overall, completeness of the physical assessment, calculated by how many components of the standardized physical assessment were performed, was 78% for the pre‐MPAC group. After MPAC training, this increased to 94% (*p* < .001). Only 48% of nurses had a pen lights or pupilometers pre‐MPAC and 86% had them post‐MPAC (*p* < .001). We found that 92% of staff had stethoscopes pre‐MPAC and 99% had them post‐MPAC (*p* < .001).

As the hospital's EWS relies on nursing documentation, timeliness and accuracy of documentation was also measured. Documentation was faster after MPAC. In the pre‐MPAC audits, the percentage of documentation completed within 4 h of the assessment was 64% and after MPAC this increased to 91% (*p* < .001). In the pre‐MPAC audits, 67% of the documentation matched what was performed during the physical assessment. After MPAC, accuracy increased to 95% (*p* < .001). Incidentally, the average length of time to perform a physical assessment decreased from 10.7 min to 9.84 min (*p* = .044).

**Figure 1:**
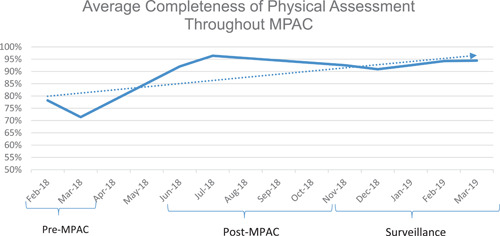
Average completeness of physical assessments throughout Methodist Proficient Assessment Competency (MPAC). [Color figure can be viewed at wileyonlinelibrary.com]

The post‐MPAC audit data collection was further separated into two phases: the post‐MPAC audits, collected for each cohort for 4 weeks starting 1 month after MPAC training, and the surveillance phase, where audits were collected for several months after MPAC training. The audits collected during the surveillance phase showed that the average completeness of physical assessments during the initial post‐MPAC period remained higher than the pre‐MPAC audits. Figure 1 demonstrates how the average completeness of physical assessments was maintained after MPAC training concluded.

RRT calls were further analyzed after MPAC as part of a research study around recognition of clinical deterioration. In a 6‐month period before MPAC training, only 23% of RRT calls occurred within 60 min of the first sign of clinical deterioration and after MPAC training this number improved to 31%. This indicates that nurses were recognizing clinical signs of deterioration sooner. Additional findings on how MPAC impacted RRT calls and improved recognition of clinical deterioration are reported in a separate publication.

## 6 DISCUSSION AND IMPLICATIONS FOR NURSING PRACTICE

All measures improved after the nurses completed MPAC. By utilizing an evidence‐based standardized physical assessment, nurses performed more complete assessments that were documented earlier and more accurately. Additionally, nurses were prepared with the appropriate assessment equipment.

Nurses are educated on how to perform a full physical assessment in their initial nursing education. However, nurses have more responsibilities than in the past and a full physical assessment was considered less important. If nursing leaders and nursing preceptors do not emphasize the importance of physical assessment, nurses focus on other priorities. During the pre‐MPAC audits, it became apparent that nurses were prioritizing physical assessments below other tasks. Because of this, when a patient's condition deteriorated, nurses were not as familiar with patient baseline information and often not prepared for the RRT if they were activated. Through MPAC, nurses were trained not only on how to do a standardized full physical assessment, but also on the importance for nursing practice and monitoring changes in patient's condition. Early recognition of subtle changes improves timeliness of RRT deployment and reduces mortality.^6^

Throughout the MPAC initiative, nurse leaders became more aware of practices surrounding physical assessment, the assessment equipment, and documentation practices. Just as staff nurses have many priorities, so do nurse leaders. Leader priorities focus more on patient satisfaction scores, hospital associated conditions, turnover, and barcode medication scanning compliance. Without a report to highlight which nurses were not carrying stethoscopes or documenting in a timely manner, other priorities took precedence. However, during the posttraining phase of the MPAC initiative, completed audits were reviewed with nurse leaders. Nurse leaders became much more aware of physical assessments and were better equipped to enforce the new standardized physical assessment and to ensure staff had the assessment equipment they needed. In the data collected from 1 month after the MPAC training through the next several months, completeness of assessments remained significantly higher than the pre‐MPAC audits and were maintained at this high level over time. This indicates that MPAC was a sustained culture change and it is likely that nurse leader engagement with physical assessments was an important component.

Another crucial measure for this project was timeliness and accuracy of assessment documentation. The EWS at this organization uses vital sign, laboratory, and nursing physical assessment data. Timely documentation of the patient's current state is necessary for the EWS to be accurate. Anytime new data are generated, the EWS score recalculates. If a nurse assesses a patient with new subtle signs of clinical deterioration but does not document the assessment for 8 h, the EWS will not reflect these changes until 8 h later. As the EWS is designed to alert healthcare providers and nurses when subtle changes could mean clinical deterioration is likely, timeliness and accuracy of documentation is crucial. During MPAC, all participants attended a learning station with case studies that demonstrated the importance of timely and accurate documentation in the EHR so the EWS would be accurate. This learning contributed to the improvement in documentation timeliness. Additionally, the legal and ethical rationale for accurate documentation were discussed, which contributed to an increase in the accuracy of documentation.

To ensure consistency during the physical assessment audits, the project leaders for MPAC had every nurse educator complete the MPAC course themselves and complete a return demonstration of the standardized physical assessment on a standardized patient before they could begin auditing. It was important that the ~20 nurse educators who contributed to the MPAC course were all comfortable with the standardized physical assessment and with consistently evaluating every nurse using the same methods and techniques every time. The educators were given time to study the course materials and to ask clarifying questions about the content and the audit process before the course began. During the MPAC training, the program leader periodically observed each educator as they manned the physical assessment station and would provide feedback to the educator if needed. If an educator was not able to provide the training as needed, they were reassigned. Similarly, during the post‐MPAC audits, the program leader would periodically observe the educators completing the audits. This contributed to consistent audit technique and created confidence in the project results.

An incidental finding was the decrease in the total time spent performing a physical assessment. The educators did not intend to shorten how long it took to complete a physical assessment, as they emphasized thoroughness over speed. During the pre‐MPAC audits, nurses were disorganized while performing a physical assessment and frequently stopped to complete other tasks. Even when they performed an incomplete assessment, it still took on average more than 10 min per patient. After MPAC, nurses were more efficient and the average time to complete their assessment decreased to 9.84 min while being more thorough.

## 7 LIMITATIONS

This quality improvement initiative had several limitations. First, the number of pre‐MPAC audits was smaller than the number of post‐MPAC audits. Even though the number of audits is different between the two groups, all inpatient units were audited in both groups on both day and night shifts. Another weakness is how the physical assessment audits were completed. As nurse educators were physically present with the nurses as they performed assessments, the nurses may have changed their usual assessment routine because they knew they were being watched. Particularly for the post‐MPAC audits, the nurse educators were the same who had witnessed return demonstrations during the MPAC classes. As such, it was not possible to determine how nurses performed the standardized physical assessment when they were not being audited. Lastly, the audit process evolved during the last 6 months of the project. Initially, the auditors just observed the nurses and did not provide any feedback. However, the nursing leadership requested that the nurse educators utilize the opportunity to provide just‐in‐time teaching should they observe something that needed coaching; so, although the same data were collected, the process evolved as time went on.

## 8 CONCLUSION

As healthcare providers who provide patient care around the clock, nurses are responsible for identifying changes in a patient's condition and taking action to prevent clinical deterioration. Performing a standardized physical assessment at the beginning of their shift allows nurses to know their patient's current status. Timely and accurate assessment documentation in the patient's EHR then facilitates EWS effectiveness. This quality improvement initiative focused on retraining nurses on how to perform an evidence‐based, standardized full physical assessment, which resulted in significant improvement in completeness of assessments with more timely and accurate documentation.

## CONFLICTS OF INTEREST

The authors declare no conflicts of interest.

## Data Availability

The data that support the findings of this quality improvement initiative are available on request from the corresponding author. The data are not publicly available due to privacy restrictions.
